# Factors associated with mental health service utilization among chronically ill minority older adults in Thailand's remote highland regions

**DOI:** 10.1016/j.pmedr.2026.103443

**Published:** 2026-03-13

**Authors:** Supaporn Sudnongbua, Samran Chuamuangphan

**Affiliations:** a Faculty of Public Health, Naresuan University, Phitsanulok Province 65000, Thailand; b Chiang Rai Provincial Health Office, Chiang Rai Province 57000, Thailand

**Keywords:** Accessibility, Mental health services, Older adults, Ethnic minorities, Psychosocial resources, Social support, Chronic conditions

## Abstract

**Objective:**

This study investigated factors associated with access to mental health services among older adults from ethnic minority groups with chronic conditions living in remote highland areas.

**Methods:**

A cross-sectional survey was conducted among 468 ethnic minority older adults with chronic conditions. Data were collected between January 8, 2024 and October 10, 2024. Pearson correlation and multiple linear regression analyses were used to identify factors associated with mental health service accessibility.

**Results:**

Multiple linear regression identified factors associated with accessibility of mental health services. Lower accessibility was associated with age ≥ 70 years, Christian religion, employment, government officer health benefit scheme, higher Thai Geriatric Mental Health scores, and reliance on ethnic-belief mental health care. Higher accessibility was associated with financial security, overall social support, female gender, Muslim religion, higher education, living with children and grandchildren, technology access, emotional and instrumental support, and personal and community-level mental health promotion. The final model explained 87% of the variance in accessibility (adjusted R^2^ = 0.86).

**Conclusion:**

Accessibility to mental health services among ethnic minority older adults is shaped by sociodemographic, cultural, psychosocial, and support-related factors. Culturally responsive and community-based strategies that strengthen instrumental support and address mental health needs are essential to improve equitable access.

## Highlights

• Accessibility varied by sociodemographic factors.
• Cultural beliefs and traditional care were linked to service accessibility.
• Financial security and technology access improved mental health service accessibility.
• Emotional and instrumental social support strongly increased service accessibility.
• Mental health promotion at personal, family, and community levels enhanced access.

## 1 Introduction

Population ageing is a major global trend, with adults aged ≥60 years projected to increase from 1.0 billion in 2020 to 2.1 billion by 2050, and the ≥80-year population expected to triple (World Health Organization, 2021; World Health Organization, 2022; World Health Organization, 2025a; United Nations and Department of Economic and Social Affairs, 2022). While increased longevity reflects public health success, it also poses major challenges for health systems (Beard et al., 2016). Older adults face a high burden of multimorbidity, noncommunicable diseases, and mental health problems, shaped by social determinants such as education, socioeconomic status, and social support (Prince et al., 2015; Courtin and Knapp, 2017; Santini et al., 2020; World Health Organization, 2023; World Health Organization, 2025b). These challenges are more pronounced among minority and indigenous older adults in remote areas, where structural barriers limit access to culturally appropriate health and mental health services (Gracey and King, 2009; Walker et al., 2017). In Thailand, highland ethnic minority older adults experience compounded disadvantages due to geographic isolation, poverty, limited health and digital infrastructure, low health literacy, and weak community-based mental health promotion, resulting in reduced access to mental health care (Techatraiphum et al., 2016; Leigh-Hunt et al., 2017; World Health Organization, 2025c).

Addressing these disparities requires policy and research approaches that integrate social determinants into health systems, consistent with the World Health Organization (WHO)’s *Jakarta Declaration on Leading Health Promotion into the 21st Century* (World Health Organization, 1997). Conceptual frameworks such as Penchansky and Thomas's Five Dimensions of Access—availability, accessibility, accommodation, affordability, and acceptability—guided the way this study conceptualized and examined factors related to mental health service utilization among chronically ill minority older adults in Thailand's remote highland regions. Each dimension informed the selection of variables relevant to structural and cultural barriers experienced by this population. For example, availability and accessibility shaped our focus on geographic and resource constraints; accommodation and affordability guided the inclusion of service organization and financial factors; and acceptability informed our attention to cultural and linguistic influences. By aligning the study's measures with this framework, we were able to interpret how multiple dimensions of access collectively influence service use in underserved highland communities.

## 2 Methods

### 2.1 Study design and population

We employed a cross-sectional study design focusing on elderly ethnic minority individuals with chronic conditions residing in remote highland areas of Thailand. Data were collected through structured interviews using a standardized questionnaire, and participants were selected through simple random sampling to enhance the representativeness of the study sample. This cross-sectional study was conducted between **January 8, 2024 and October 10, 2024**.

The study assessed multiple determinants of mental health service accessibility, including demographic characteristics, living arrangements, educational attainment, financial security, technology access, interpersonal relationships, psychosocial resources, social supports, methods of mental health care, and participation in mental health promotion activities. Multiple regression analyses were conducted to identify significant associated factors of mental health service accessibility.

The study took place in Mae Chan District in northern Chiang Rai Province, Thailand. Participants were selected through a three-stage simple random sampling procedure that involved randomly choosing provinces and districts, then subdistricts, and finally households. Individuals were eligible if they were at least 60 years old, belonged to an ethnic minority group and/or had a chronic illness, and were cognitively capable of taking part. Ethnic minority status was classified based on categories established by the Thai Ministry of Interior, and chronic illness was defined as a physician-diagnosed condition lasting 12 months or longer, as recorded in primary care center medical records. A total of 468 participants were included, with the sample size calculated using the formula for estimating a proportion in a finite population (Lwanga and Lemeshow, 1991). The total population of interest was 32,685 individuals. Assuming a 50% expected prevalence of the outcome (to maximize sample size), a 95% confidence level (Z = 1.96), and a margin of error of 5% (d = 0.05), the required sample size was calculated as 421 participants. The formula used was:

$$n={\mathrm{NZ}}^{2}P\left(1-P\right)/d^{2}\left(N-1\right)+Z^{2}P\left(1-P\right)$$

where *n* is the sample size, *N* is the total population, *P* is the expected prevalence, *Z* is the standard normal value, and *d* is the desired precision. Participants were then selected using a **simple random sampling technique** from the population. To account for potential non-response or incomplete data, an additional 10% was added, yielding **a final sample size of 468 participants.** Among the randomly selected participants, 45.50% self-identified as Akha (*n* = 213), 20.30% as Shan (*n* = 95), 16.70% as Lahu (*n* = 78), and 17.50% as Lisu (Muser) (*n* = 82).

### 2.2 Ethical considerations

The study adhered to the Declaration of Helsinki and relevant national human research regulations, with ethical approval from the Institutional Review Board of the Chiang Rai Provincial Health Office (IRB-CRPHO) (Approval No. CRPHO 225/2566). Participants were fully informed about the study and provided written consent, with the right to withdraw at any time. Confidentiality and anonymity were maintained, and special care ensured that vulnerable populations, including ethnic minorities and older adults, could give informed consent in a culturally and linguistically appropriate manner.

### 2.3 Measures

The survey comprised five major components corresponding to the variables included in the regression analyses: (1) sociodemographic and cultural characteristics; (2) health benefit schemes, health care practices, and sources of health information; (3) psychosocial resources; (4) social support and mental health–related measures; and (5) accessibility of mental health services, which served as the outcome variable.

#### 2.3.1 Sociodemographic, cultural, and health-related characteristics

Structured, face-to-face interviews were conducted using a standardized questionnaire to obtain participants' sociodemographic information. Variables included gender, age, marital status, religion, cultural background and beliefs, living arrangements, educational attainment, employment status, type of health benefit scheme, health care practices, and sources of health information. These variables were included as covariates in the regression analyses.

#### 2.3.2 Psychosocial resources

Four psychosocial resource domains were assessed based on participants' experiences during the past 12 months: technology access, interpersonal relationships, financial security, and social support. The psychosocial resource framework was adapted from Turner and Roszell (1994), and items were refined through expert review.

Technology access (13 items) and interpersonal relationships (12 items) were measured using a 5-point Likert scale (1 = “never,” 2 = “rarely,” 3 = “sometimes,” 4 = “often,” 5 = “always”). Internal consistency reliability in the present study was strong (Cronbach's α = 0.88). Mean scores ranged from 1.00 to 5.00, with higher values indicating greater access.

Financial security was assessed using 12 items with the same response format (Cronbach's α = 0.88). These items reflected employment status, secondary income-generating activities, interest in livelihood training, adequacy of income for daily needs, reliance on children or grandchildren for financial support, savings for daily expenses, regular debt obligations, and financial capacity to cover medical expenses and emergencies.

#### 2.3.3 Social support

Social support was evaluated using the Thai Social Support Questionnaire (SSQ) (Sudnongbua, 2011), which measures emotional, financial, material, and instrumental support. Items were rated on a 5-point Likert scale consistent with other psychosocial measures. The scale demonstrated strong internal consistency in the present study (Cronbach's α = 0.90).

Both an overall social support score and four subdomain scores (emotional, financial, material, and instrumental support) were computed. The overall score was entered in Model 1 of the regression analysis, while the four subdomain scores were entered separately in Model 2 to examine the differential effects of specific types of social support.

#### 2.3.4 Mental health–related measures

Mental health status was assessed using the Thai Geriatric Mental Health Assessment Tool (T-GMHA-15) (Department of Mental Health, Ministry of Public Health of Thailand, 2025). The 15-item instrument evaluates emotional symptoms such as anxiety, sadness, loneliness, irritability, and sleep problems based on experiences within the past 12 months. Items are rated on a 4-point Likert scale (1 = “not at all,” 2 = “a little,” 3 = “moderately,” 4 = “extremely”). The instrument demonstrated excellent internal consistency in the current sample (Cronbach's α = 0.96). Higher mean scores indicate better mental health well-being.

Mental health promotion was assessed using a 13-item questionnaire adapted from *Mental health promotion for elderly populations in the World Health Organization South-East Asia Region* (Pandey et al., 2022). The scale covers individual, family, and community domains and uses a 4-point Likert response format (0 = “not at all” to 3 = “extremely”). Internal consistency reliability in the present study was strong (Cronbach's α = 0.89). Higher scores indicate greater engagement in mental health promotion activities.

#### 2.3.5 Accessibility of mental health services (outcome variable)

Accessibility of mental health services was assessed using 10 items developed from a literature review guided by the World Health Organization (WHO) framework on social determinants of mental health (World Health Organization, 2025b). The items measured experiences of accessing mental health services during the past 12 months on a 5-point Likert scale (1 = “never” to 5 = “always”).

Accessibility was conceptualized as perceived ease of obtaining services, encompassing availability, affordability, and acceptability. Item 10 was reverse-coded. The overall score was calculated as the mean of all items, with higher scores indicating greater perceived accessibility. Internal consistency was strong (Cronbach's α = 0.88).

### 2.4 Statistical analysis

Descriptive statistics, including proportions, means, and standard deviations, were used to summarize the data. Categorical variables were dichotomized and analyzed using point-biserial correlation coefficients, while Pearson's product–moment correlation was employed to examine associations between key factors and accessibility of mental health services among elderly ethnic minority individuals with chronic conditions.

Standard multiple regression analyses were conducted using two models to identify factors associated with accessibility. Independent variables comprised a combination of ordinal and categorical measures. Ordinal variables were analyzed according to their original ordered response categories, whereas categorical variables were entered into the regression models using dummy coding with clearly specified reference categories. Selected variables were dichotomized where appropriate to enhance model stability.

Two multiple linear regression models were constructed. Model 1 included sociodemographic characteristics, cultural factors, health benefit schemes, health care practices, sources of health information, psychosocial resources, and mental health variables, including overall social support. Model 2 replaced overall social support with its four subdomains (emotional, financial, material, and instrumental support) to examine the differential effects of specific types of social support on accessibility of mental health services. This approach was adopted to reduce multicollinearity and to assess whether specific dimensions of social support provided greater explanatory value than the overall score.

Multicollinearity was assessed using variance inflation factors (VIF) and tolerance statistics, with VIF values greater than 5 indicating problematic multicollinearity. Accordingly, overall social support and its four subdomains were not entered into the same model simultaneously; overall social support was retained in Model 1, whereas the four subdomains were included in Model 2. In addition, the social security scheme was excluded due to high collinearity with traditional medicine care, living with a spouse was excluded due to collinearity with marital status, medical care was excluded due to collinearity with ethnic-belief care, and formal mental health care was excluded due to collinearity with no mental health care (all VIF > 5).

Statistical significance was set at *p* < 0.05 (two-tailed). All analyses were performed using Statistical Package for the Social Sciences (SPSS) Statistics for Mac, Version 23.0.

## 3 Results

### 3.1 Sociodemographic and health factors associated with mental health service accessibility

The sociodemographic characteristics of the study participants were assessed to provide a comprehensive overview of their background and context. Table 1 presents the associations between sociodemographic, cultural, and health-related factors and accessibility of mental health services.

**Table 1:** Correlations between sociodemographic, cultural, and health-related factors and accessibility of mental health services among older adults in remote highland regions of Thailand, January–October 2024 (*n* = 468).

Variables	n (%)	Mean	S.D.	r	*p*-value
Gender					
Male	216 (46.20)	0.46	0.50	0.02	0.69
Female	252 (53.80)	0.54	0.50	−0.02	0.69

Age					
60–69	354 (75.60)	0.76	0.43	0.32	< 0.01**
70 and over	114 (24.40)	0.24	0.43	−0.32	< 0.01**

Marital status					
Not married	259 (55.30)	0.55	0.50	−0.17	< 0.01**
Married	209 (44.70)	0.45	0.50	0.17	< 0.01**

Religion					
Buddhist	388 (82.90)	0.83	0.38	0.02	0.62
Christian	34 (7.30)	0.07	0.26	−0.07	0.16
Muslim	46 (9.80)	0.10	0.30	0.03	0.54

Culture and belief					
No belief	239 (51.10)	0.51	0.50	0.26	< 0.01**
Belief in spirits	208 (44.40)	0.44	0.50	−0.14	< 0.01**
Belief in supernatural beings	21 (4.50)	0.04	0.21	−0.30	< 0.01**

Living arrangements					
Living alone	68 (14.50)	0.15	0.35	−0.21	< 0.01**
Living with spouse	191 (40.80)	0.41	0.49	0.47	< 0.01**
Living with children and grandchildren	209 (44.70)	0.45	0.50	−0.31	< 0.01**

Education					
Low education	413 (88.30)	0.88	0.32	−0.29	< 0.01**
Secondary or higher	55 (11.70)	0.12	0.32	0.29	< 0.01**

Employment status					
Not employed	222 (47.40)	0.47	0.50	−0.19	< 0.01**
Employed	246 (52.60)	0.53	0.50	0.19	< 0.01**

Health benefit scheme					
Universal coverage scheme	420 (89.80)	0.90	0.30	−0.34	< 0.01**
Social security scheme	24 (5.10)	0.05	0.22	0.51	< 0.01**
Government officer scheme	24 (5.10)	0.05	0.22	−0.05	0.27

Methods of physical health care					
None of care / Let it be	9 (1.90)	0.02	0.14	−0.04	0.41
Ethnic-belief care	20 (4.30)	0.04	0.20	0.23	< 0.01**
Medical care	439 (93.80)	0.94	0.24	−0.17	< 0.01**

Methods of mental health care					
None of care / Let it be	173 (37.00)	0.37	0.48	−0.41	< 0.01**
Ethnic-belief care	21 (4.50)	0.04	0.21	−0.04	0.43
Traditional medicine care	34 (7.30)	0.07	0.26	0.55	< 0.01**
Formal mental health care	240 (51.20)	0.52	0.50	0.12	0.01**

Sources of health information					
Broadcasting tower of village	87 (18.60)	0.19	0.39	0.33	< 0.01**
Health volunteers	317 (67.70)	0.68	0.47	−0.20	< 0.01**
Government brochure/poster	30 (6.40)	0.06	0.25	−0.05	0.24
Broadcasting car	34 (7.30)	0.07	0.26	−0.09	0.05*

*Note.* Data are presented as n (%) for categorical variables and mean ± standard deviation (S.D.). Pearson correlation coefficients with accessibility to mental health services are presented as “**r**”. Accessibility of mental health services was the dependent variable. For dichotomous categorical variables, point-biserial correlation coefficients were calculated. For ordinal or continuous variables, Pearson's correlation coefficient was used. *p* < 0.05 was considered statistically significant (*p* ≤ 0.05 = *, *p* ≤ 0.01 = **).

The sample consisted of slightly more females (53.80%) than males (46.20%). Most participants were aged 60–69 years (75.60%), married (44.70%), Buddhist (82.90%), and had a low level of education (88.30%). Over half of the participants were employed (52.60%) and the majority were covered by the universal health coverage scheme (89.80%). Regarding living arrangements, most lived with children and grandchildren (44.70%) or with a spouse (40.80%). Medical care was the most common method of physical health care (93.80%), and just over half of the participants used formal mental health care services (51.20%). Health volunteers were the main source of health information (67.70%).

Correlation analysis indicated that **age, marital status, culture and belief, living arrangements, education, employment status, health benefit scheme, methods of physical and mental health care, and sources of health information** were significantly associated with accessibility of mental health services (*p* ≤ 0.05). In contrast, **gender and religion** showed no statistically significant associations with accessibility (*p* > 0.05).

Overall, the table demonstrates that accessibility of mental health services among older adults is more strongly related to social, cultural, and structural factors than to basic demographic characteristics such as gender or religion.

### 3.2 Associations of psychosocial and mental health factors with service accessibility

Psychosocial resources, including financial security, accessibility of technology, and interpersonal relationships, as well as multiple components of social support, were examined for their association with accessibility. Mental health promotion was assessed at the personal, family, and community levels, and overall T-GMHA-15 scores were included to evaluate geriatric mental health status in relation to service accessibility (see Table 2).

**Table 2:** Correlations between psychosocial resources, social support, Thai Geriatric Mental Health, mental health promotion, and accessibility of mental health services among older adults in remote highland regions of Thailand, January–October 2024 (n = 468).

Variables	Mean	S.D.	r	p-value
Accessibility of mental health services (Dependent variable)	3.03	0.48		

Psychosocial resources				
1. Financial security	3.28	0.30	0.32	p < 0.01**
2. Accessibility of technology	3.08	0.73	0.16	*p* < 0.01**
3. Interpersonal relationship	3.39	0.64	0.18	p < 0.01**

Social Supports (4 components)				
1. Emotional support	2.46	0.61	0.14	p < 0.01**
2. Financial support	2.48	0.57	0.36	p < 0.01**
3. Material support	2.60	0.53	0.37	*p* < 0.01**
4. Instrumental support	2.62	0.59	0.56	p < 0.01**
Overall social supports	2.54	0.45	0.46	p < 0.01**
Thai Geriatric Mental Health Assessment Tool (T-GMHA-15)	3.20	0.32	−0.16	p < 0.01**

Mental health promotion				
1. Personal level of mental health promotion	3.09	0.39	0.25	p < 0.01**
2. Family level of mental health promotion	3.59	0.54	−0.08	0.08
3. Community level of mental health promotion	2.64	0.58	0.44	p < 0.01**
Overall mental health promotion	3.87	0.31	0.32	p < 0.01*

*Note.* Variables are reported as mean and standard deviation (SD). Pearson correlation coefficients with accessibility to mental health services are presented as “**r**”. Accessibility to mental health services served as the dependent variable. Associations were examined using Pearson's correlation analysis. Statistical significance was set at p < 0.05 (* p < 0.05, ** *p* < 0.01).

The mean accessibility score was 3.03 (SD = 0.48), indicating moderate access to mental health services. Accessibility was positively correlated with psychosocial resources, particularly financial security, and weakly with technology access and interpersonal relationships. All social support components were significantly associated with accessibility, with instrumental support showing the strongest correlation, followed by overall, financial, and material support. Poorer mental health status (higher T-GMHA-15 scores) was negatively correlated with accessibility. Personal and community-level mental health promotion were positively associated with accessibility, whereas family-level promotion was not significant. Overall, greater psychosocial resources, stronger social support, and higher mental health promotion were linked to better accessibility, while poorer mental health was linked to reduced access.

### 3.3 Factors associated with accessibility of mental health services

This section examined factors associated with the accessibility of mental health services among older adults from ethnic minority groups living with chronic conditions. Understanding these factors is critical for identifying associated factors of accessibility and for informing strategies to improve service delivery and utilization among vulnerable populations (see Table 3).

**Table 3:** Multiple linear regression analysis of factors associated with accessibility of mental health services among older adults in remote highland regions of Thailand, January–October 2024 (n = 468).

Variable	Model 1		Model 2	
Dependent Variable: Accessibility of mental health services				
Constant	B(SE)	p-value	B(SE)	p-value
Gender (Ref. = Male)				
Female	0.03(0.03)	0.26	0.06 (0.03)	0.02*
Age (Ref. =60–69 years old)				
Age 70 and over	−0.06(0.03)	0.05*	−0.13(0.03)	p < 0.01**
Religion (Ref. = Buddhist)				
Christian	−0.42(0.10)	p < 0.01**	−0.36(0.08)	p < 0.01**
Muslim	0.07(0.06)	0.21	0.25(0.05)	p < 0.01**
Culture and belief (Ref. = No belief)				
Belief in spirits	0.03(0.04)	0.47	−0.23(0.04)	p < 0.01**
Belief in supernatural beings	0.25(0.13)	0.06	0.03(0.11)	0.79
Living arrangements (Ref. = Living alone)				
Living with children and grandchildren	0.08(0.04)	0.07	0.09(0.03)	p < 0.01**
Education (Ref. = low education)				
Secondary or higher	0.04(0.05)	0.49	0.16(0.04)	p < 0.01**

Employment status (Ref. = Not employed)				
Employed	−0.08(0.03)	0.03*	−0.07(0.03)	0.03*

Health benefit scheme (Ref. = Universal coverage scheme)				
Government officer scheme	−0.16(0.06)	0.01**	−0.22(0.05)	p < 0.01**

Methods of physical health care (Ref. = None of care/Let it be)				
Ethnic-belief care	0.27(0.08)	p < 0.01**	0.35(0.07)	p < 0.01**
Methods of mental health care (Ref. = None of care/Let it be)				
Ethnic-belief care	−0.24(0.08)	p < 0.01**	−0.40(0.06)	p < 0.01**
Traditional medicine care	0.60(0.08)	p < 0.01**	0.42(0.07)	p < 0.01**
Sources of health information (Ref. = Health volunteers)				
Broadcasting Tower of village	−0.04(0.05)	0.46	−0.04(0.05)	0.33
Government brochure/poster	−0.39(0.07)	p < 0.01**	−0.36(0.05)	p < 0.01**
Broadcasting car	0.23(0.06)	p < 0.01**	0.41(0.05)	p < 0.01**
Financial security	0.39(0.05)	p < 0.01**	0.39(0.05)	p < 0.01**
Accessibility of technology	0.01(0.03)	0.74	0.21(0.03)	p < 0.01**
Interpersonal relationship	−0.08(0.03)	0.01**	−0.15(0.03)	p < 0.01**
Thai Geriatric Mental Health Assessment Tool (T-GMHA-15)	−0.37(0.05)	p < 0.01**	−0.15(0.05)	p < 0.01**
Personal level of mental health promotion	0.33(0.04)	p < 0.01**	0.14(0.04)	p < 0.01**
Family level of mental health promotion	−0.24(0.03)	p < 0.01**	−0.24(0.03)	p < 0.01**
Community level of mental health promotion	0.34(0.03)	p < 0.01**	0.19(0.03)	p < 0.01**
Overall social support	0.63(0.04)	p < 0.01**		
Emotional support			0.14(0.02)	p < 0.01**
Financial support			−0.13(0.04)	p < 0.01**
Material support			0.02(0.04)	0.59
Instrumental support			0.61(0.04)	p < 0.01**

Model Statistics				
R^2^	0.82		0.87	
Adjusted R^2^	0.81		0.86	
F	77.94**		107.67**	
df	(25, 438)		(27, 436)	
N	463		463	

*Note.* Unstandardized regression coefficients (B) are reported, with standard errors (SE) in parentheses. *p ≤ 0.05 ** *p* ≤ 0.01.

Model 1 included overall social support as a composite score, whereas Model 2 replaced the overall score with its four subdomains (emotional, financial, material, and instrumental support). Overall social support and its subdomains were not entered simultaneously to avoid multicollinearity. The following variables were excluded due to high collinearity (VIF > 5): social security scheme (collinear with traditional medicine care), living with a spouse (collinear with marital status), medical care (collinear with ethnic-belief care), and formal mental health care (collinear with no mental health care).

Accessibility of mental health services was influenced by demographic, cultural, health-system, psychosocial, and social support factors. In Model 1, lower accessibility was associated with older age, Christian religion, employment, government officer health benefit coverage, ethnic-belief mental health care, government information sources, stronger interpersonal relationships, and poorer mental health, while higher accessibility was linked to traditional practices, community-based information, financial security, mental health promotion, and overall social support (Adjusted R^2^ = 0.81). After adjustment in Model 2, higher accessibility remained associated with female gender, Muslim religion, co-residence with family, higher education, financial security, technology access, personal and community mental health promotion, and emotional and instrumental support, whereas older age, certain beliefs, health system factors, poorer mental health, family-level promotion, and financial support were linked to lower accessibility. Model 2 showed improved explanatory power (Adjusted R^2^ = 0.86).

## 4 Discussion

Sociodemographic, cultural, psychosocial, and structural factors shaped mental health service accessibility among ethnic minority older adults. Higher education was consistently associated with better access, likely reflecting improved health literacy and ability to navigate mental health systems (Meyer et al., 2025; Travers et al., 2022). Older age (≥70 years) was negatively associated, suggesting that mobility limitations, dependency, and reduced awareness hinder access (Hao et al., 2025). Females demonstrated higher accessibility, potentially reflecting stronger help-seeking behaviors. Living with children and grandchildren was negatively associated, indicating that dependence on household members may constrain independent service use (Reynolds et al., 2022; Wu et al., 2025).

Cultural beliefs and health practices were important determinants. Use of ethnic-belief care for physical health and traditional medicine for mental health facilitated access, suggesting that culturally familiar practices may serve as entry points to formal services (Jimenez et al., 2012; Cabañero-Garcia et al., 2025). Conversely, reliance on ethnic-belief mental health care alone was negatively associated with accessibility, potentially reflecting delayed help-seeking. These findings emphasize the importance of culturally sensitive and integrative approaches that link traditional and formal care (Chen et al., 2025; Elshaikh et al., 2023).

Psychosocial resources and social support were strongly related to accessibility. Financial security consistently improved access by reducing structural barriers (Dong et al., 2024; Reynolds et al., 2022), and technology access facilitated service use. Instrumental support emerged as the strongest predictor, highlighting the role of practical assistance such as transportation and accompaniment (Yiu et al., 2025; Valtorta et al., 2018). Emotional support contributed positively, whereas financial support alone was negatively associated, suggesting that practical or motivational support is more critical than monetary aid (Dong et al., 2024).

Mental health promotion further influenced access. Personal- and community-level promotion were positively associated, reflecting the benefits of proactive engagement and community initiatives (Reynolds et al., 2022). Family-level promotion was negatively associated, possibly reflecting overreliance on family coping that delays formal service use. Higher T-GMHA-15 scores were consistently linked to lower accessibility, indicating the need for targeted outreach and early intervention (Ikeda et al., 2025; Wu et al., 2025).

Overall, accessibility is shaped by the interplay of demographic, cultural, psychosocial, and health system factors. Improving access requires culturally responsive strategies, strengthened instrumental and technological support, and proactive engagement with those experiencing poorer mental health (Elshaikh et al., 2023). However, due to the cross-sectional design, findings should be interpreted as associative rather than causal.

## 5 Conclusion

Accessibility to mental health services among older ethnic minority adults is shaped by demographic, cultural, psychosocial, and structural factors, with better access linked to financial security, community-based resources, traditional practices, and instrumental support, highlighting the need for culturally sensitive, community-based strategies.

## CRediT authorship contribution statement

**Supaporn Sudnongbua:** Writing – review & editing, Writing – original draft, Visualization, Validation, Supervision, Software, Resources, Project administration, Methodology, Investigation, Formal analysis, Data curation, Conceptualization. **Samran Chuamuangphan:** Writing – review & editing, Writing – original draft, Visualization, Validation, Supervision, Resources, Methodology, Investigation, Data curation, Conceptualization.

## Funding

None received.

## Declaration of competing interest

The authors declare that they have no known competing financial interests or personal relationships that could have appeared to influence the work reported in this paper.

## Data Availability

Data will be made available on request.
